# Acid Adaptation Enhances Tolerance of *Escherichia coli* O157:H7 to High Voltage Atmospheric Cold Plasma in Raw Pineapple Juice

**DOI:** 10.3390/microorganisms12061131

**Published:** 2024-06-01

**Authors:** Allison Little, Aubrey Mendonca, James Dickson, Paulo Fortes-Da-Silva, Terri Boylston, Braden Lewis, Shannon Coleman, Emalie Thomas-Popo

**Affiliations:** 1 Department of Food Science and Human Nutrition, Iowa State University, Ames, IA 50011, USA; alittle1@iastate.edu (A.L.); paulo@iastate.edu (P.F.-D.-S.); tboylsto@iastate.edu (T.B.); bjlewis@iastate.edu (B.L.); scoleman@iastate.edu (S.C.); emaliepopo@gmail.com (E.T.-P.); 2 Interdepartmental Microbiology Program, Iowa State University, Ames, IA 50011, USA; jdickson@iastate.edu; 3 Department of Animal Science, Iowa State University, Ames, IA 50011, USA; 4 Department of Biochemistry, Biophysics & Molecular Biology, Iowa State University, Ames, IA 50011, USA

**Keywords:** acid adaptation, *Escherichia coli* O157:H7, cold plasma, pineapple juice, sub-lethal injury

## Abstract

Pathogens that adapt to environmental stress can develop an increased tolerance to some physical or chemical antimicrobial treatments. The main objective of this study was to determine if acid adaptation increased the tolerance of *Escherichia coli* O157:H7 to high voltage atmospheric cold plasma (HVACP) in raw pineapple juice. Samples (10 mL) of juice were inoculated with non-acid-adapted (NAA) or acid-adapted (AA) *E. coli* to obtain a viable count of ~7.00 log_10_ CFU/mL. The samples were exposed to HVACP (70 kV) for 1–7 min, with inoculated non-HVACP-treated juice serving as a control. Juice samples were analyzed for survivors at 0.1 h and after 24 h of refrigeration (4 °C). Samples analyzed after 24 h exhibited significant decreases in viable NAA cells with sub-lethal injury detected in both NAA and AA survivors (*p* < 0.05). No NAA survivor in juice exposed to HVACP for 5 or 7 min was detected after 24 h. However, the number of AA survivors was 3.33 and 3.09 log_10_ CFU/mL in juice treated for 5 and 7 min, respectively (*p* < 0.05). These results indicate that acid adaptation increases the tolerance of *E. coli* to HVACP in pineapple juice. The potentially higher tolerance of AA *E. coli* O157:H7 to HVACP should be considered in developing safe juice processing parameters for this novel non-thermal technology.

## 1. Introduction

The global market for fresh fruit and juice has grown consistently in the last two decades. For fresh fruit, the market was valued at USD 551.1 billion in 2021 and has a projected compound annual growth rate (CAGR) of 3.6% from 2022 to 2028 [1]. Likewise, the fresh juice market is also growing with a higher forecasted CAGR of 8.55% between 2022 and 2027, and a projected increase in market size to USD 104.21 billion [2]. Part of the increase in the popularity of fresh fruit and juice is the plethora of health benefits that consumers derive from their consumption [3]. While there is an increasing demand for minimally processed fruit and fruit juice, these nutritious products may contain pathogenic bacteria if they are not properly handled during harvesting or in food processing establishments [4].

Unpasteurized fruit juice can harbor pathogenic bacteria, such as *E. coli* O157:H7 in apple juice and *Salmonella* spp. in orange juice [5]. Fruit is mainly grown in orchards where they are exposed to many sources of microbial contamination, such as water, windblown dust, insects, birds, and feral animals [6,7]. While whole fresh fruit is cleaned and cooled using water baths and dump tanks, these methods can sometimes lead to whole fruit being infiltrated by bacteria in the wash water [8,9]. Pathogenic bacteria enter fruit juice when the contaminated fruit is pressed to release the juice. Another mode of microbial access to fresh juice is via the transfer of microorganisms from the surface of whole fruit to the flesh during peeling and slicing or from the processing environment [10]. Enteric pathogens may survive long enough in fruit juice to pose a food safety risk to consumers. For example, *E. coli* O157:H7 and *Salmonella* spp. survived in refrigerated (4.4 °C) pineapple juice for over 42 days [11].

Deadly outbreaks of *E. coli* 0157:H7 in unpasteurized apple juice in the 1990s resulted in the U.S. Food and Drug Administration (FDA) issuing a juice HACCP (Hazard Analysis Critical Control Points) regulation. According to that regulation, juice manufacturers must implement a kill step in juice processing that produces a 5-log (99.999%) reduction of the most pertinent microorganism [5,12]. Thermal pasteurization can achieve a 5-log reduction; however, thermal treatments can destroy heat-sensitive nutrients and bioactive components in juices [13]. Moreover, thermal processing can alter the quality attributes of juice, such as color and flavor [14]. Due to these challenges, juice manufacturers have developed a keen interest in non-thermal technologies to treat juice. Such technologies include high-pressure processing, pulsed electric fields, ultraviolet light, ultrasound, and cold plasma [15,16,17,18].

Atmospheric cold plasma (ACP) is a novel non-thermal technology that utilizes the fourth state of matter (plasma) to inactivate microorganisms in foods [18,19]. Gaseous plasma consists of a mixture of electrons, positive and negative ions, excited atoms and molecules, gas atoms, free radicals, UV photons, and visible light [20]. These reactive species interact with the oxygen and nitrogen gas in air to form several reactive oxygen and nitrogen species (RONS), such as atomic oxygen, hydroxyl radical (∙OH), nitric oxide (NO), and nitrogen dioxide (NO_2_). These RONS in plasma are largely responsible for the antimicrobial effects of cold plasma [21,22]. One advantage of ACP is that it can inactivate microorganisms without high temperatures [21]. The antimicrobial efficacy of ACP against foodborne microorganisms is well documented [9,23,24,25]. However, antimicrobial efficacy may be lessened following the prior exposure of microorganisms to environmental stress [26].

Foodborne microorganisms inevitably encounter environmental stress during food production, manufacturing, storage, distribution, and preparation. Pathogens’ response to stress may cause stress adaptation and an increase in pathogen tolerance to single or multiple stressors [27,28]. For example, acid adaptation can enhance the survival of pathogens in fruit juice to pose a food safety risk to consumers. More importantly, acid-adaption may cross-protect pathogens against various processing treatments, such as heating, and non-thermal treatments [29,30,31,32,33]. The increased tolerance of stress-adapted pathogens to certain non-thermal processing treatments raises concerns about the overestimation of antimicrobial treatments, especially when non-stress-adapted organisms are used in process validation studies. Moreover, non-thermal physical treatments, depending on their severity, can cause sub-lethal injury in pathogen survivors [34].

Sub-lethally injured foodborne pathogens pose an insidious problem for food processors. If they are undetected in foods, they may resuscitate under suitable conditions and regain their pathogenicity [35]. Traditionally, the plating of diluted food samples on both selective and non-selective agar has been used to evaluate the extent of sublethal injury in pathogen survivors of an antimicrobial treatment [35,36,37,38]. Selective agar media allow growth of non-injured pathogens but inhibit resuscitation of sub-lethally injured pathogens, thus preventing their detection. Nonselective agar permits the enumeration of both non-injured and sub-lethally injured pathogens [35]. In this regard, the difference in bacterial colony counts on non-selective and selective media is used in evaluating the percent sub-lethal injury in the survivors.

While there is a growing body of knowledge on the effectiveness of non-thermal technologies for inactivating foodborne pathogens, published reports on the tolerance of stress-adapted pathogens to those technologies are scarce. To our knowledge, except for two reports [15,39], there is no published research on the tolerance of AA pathogens to ACP. Accordingly, the main objective of the present research was to evaluate the tolerance of AA *E. coli* O157:H7 to HVACP in pineapple juice. A secondary objective was to determine the extent of sub-lethal injury in both NAA and AA survivors of that pathogen following HVACP treatment of pineapple juice.

## 2. Materials and Methods

### 2.1. Bacterial Strains and Culture Conditions

Shiga-toxin-producing *Escherichia coli* O157:H7 (FRIK 125), isolated from an outbreak linked to apple cider, was obtained from Dr. Charles Kaspar, University of Wisconsin. Frozen stock cultures (−80 °C) in brain heart infusion (BHI) broth (Difco; Becton Dickinson, Sparks, MD, USA) with added glycerol (10% *v*/*v*) were thawed under cold running water and activated in tryptic soy broth supplemented with 6.0 g/L yeast extract (TSBYE; pH 7.2; Difco; Becton Dickinson) at 35 °C. Working cultures were held at 4 °C until use in the experiments. Two consecutive 24-h transfers of a working culture in tryptic soy broth without dextrose (TSB − G) and in TSB supplemented with 7.5 g dextrose per liter (TSB + G) (35 °C) were performed to obtain non-acid adapted (NAA) and acid-adapted (AA) cells, respectively [29]. The final pH values for NAA and AA cultures were 7.0–7.2 and 4.6–4.8, respectively.

### 2.2. Preparation of Inocula

For NAA and AA *E. coli* O157:H7, equal volumes (1.5 mL) of each cell type were aseptically transferred to 2-mL microcentrifuge tubes. Cells were harvested by centrifugation (10,000× *g*, 10 min, 22 °C) using a Beckman Coulter Microfuge 16 Centrifuge (Beckman Coulter, Inc., Brea, CA, USA). The pelleted cells were suspended in 1.5 mL of NaCl (8.5 g/L; saline) to yield a concentration of ~9.0 log_10_ colony-forming units (CFU)/mL as verified by plate counts on tryptic soy agar supplemented with 6 g/L yeast extract (TSAYE). Colony counts of NAA and AA *E. coli* O157:H7 were determined by serial diluting (10-fold) cell suspensions in saline and surface plating appropriate dilutions on selective agar (sorbitol MacConkey agar (SMAC)) and non-selective agar (TSAYE). SMAC is a selective, differential medium for detecting sorbitol-negative *E. coli*, such as serotype O157:H7, used in the present study. Bacterial colonies were counted after aerobic incubation (35 °C) of TSAYE and SMAC agar for 24 and 48 h, respectively.

### 2.3. Preparation and Inoculation of Pineapple Juice

Whole pineapples (Del Monte Gold^®^ Extra Sweet, Del Monte Fresh Produce N.A. Coral Gables, FL, USA) from the same production lot were purchased from a local grocery store in Ames, Iowa. The pineapples were rinsed with distilled water, and then the outside rind, top, bottom, and cores were removed using a clean knife and polypropylene cutting board. The flesh of the pineapples was cut into smaller chunks that were used to extract juice. The pineapple juice was extracted using a juice extractor (Model #67608Z, Hamilton Beach Big Mouth Pro Juice Extractor, Glen Allen, VA, USA). Particulates in the juice were removed by filtering the juice through two double layers of cheese cloth clamped with five 2-inch metal binder clips over a stainless steel strainer. Two hundred milliliters of the filtered juice were transferred to a sterile 250-mL Erlenmeyer flask. Ten milliliters of juice were aseptically transferred to appropriately labeled sterile Petri dishes (60 mm × 15 mm) and inoculated with 100 µL of either NAA or AA *E. coli* O157:H7 for an initial viable count of ~7.0 log_10_ CFU/mL. The inoculated juice samples with lids on were held at 22 ± 1 °C for no more than 0.5 h before exposing them (with lids off) to HVACP.

### 2.4. Treatment of Juice Samples with HVACP

**Figure 1:**
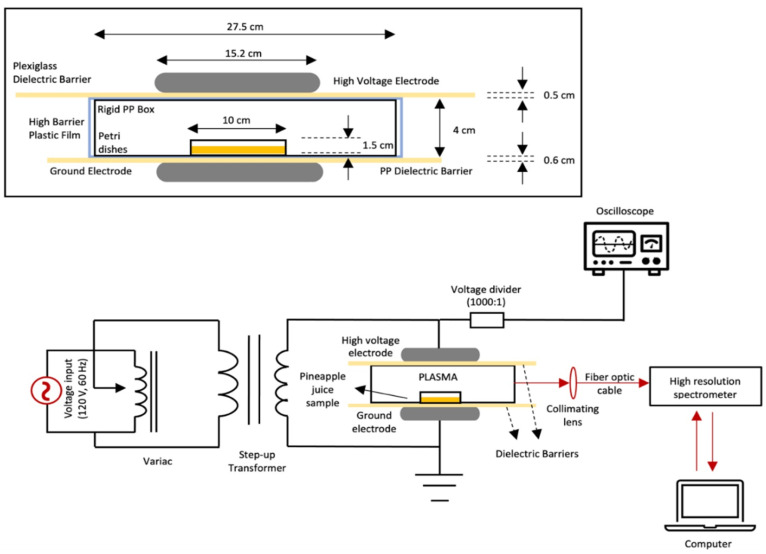
Schematic of the components of the dielectric barrier, high voltage atmospheric cold plasma (HVACP) system used to treat pineapple juice.

A schematic of the dielectric barrier discharge (DBD) HVACP system for generating atmospheric plasma is shown in Figure 1. That system produces low temperature atmospheric plasma for in-package plasma treatment [40]. An input voltage of 120 V (AC) at 60 Hz is amplified by a step-up transformer (Phenix Technologies, Inc., Accident, MD, USA). Just before applying HVACP, the lids were removed from the Petri dishes to allow direct contact of the plasma with the juice. The uncovered samples were placed in the approximate center of a rigid polypropylene box. The boxes were closed and placed in separate 35 cm × 27 cm high-barrier polypropylene Cryovac bags (B2630, Cryovac Sealed Air Corp., Duncan, SC, USA). All bags with boxes of samples were heat-sealed to retain air at atmospheric pressure. For each experiment, the relative humidity of the air was recorded. Each bag containing a box was placed between two 15.2-cm diameter aluminum electrodes with a discharge distance of 5.1 cm between the electrodes. A layer of Plexiglass was placed under the top electrode, whereas a polypropylene layer (38.5 cm × 27.3 cm) was placed above the ground electrode. The Plexiglass and polypropylene layers served as dielectric barriers to prevent arching and spark discharge [41]. The juice samples were treated with HVACP (70 kV) for 0 (control), 1, 3, 5, and 7 min. One group of samples was analyzed at ~6.0 min (0.1 h) after HVACP treatment, while the other group was analyzed after 24 h of post-treatment storage at 4 °C. The control samples (no HVACP treatment) were handled in the same way as the other samples. For physicochemical tests (pH and degrees Brix), non-inoculated samples were treated with HVACP (70 kV) for 0 (control), 3, and 7 min. Each experiment was replicated at least three times.

### 2.5. Microbial Analysis of Juice Samples

At 0.1 h and 24 h after the HVACP treatment of the juice, the bags were cut open, and Petri dishes containing the inoculated juice were removed from the boxes. Each sample was gently swirled to mix it, and 1.0 mL of juice was serially diluted (10-fold) in double-strength (2×) buffered peptone water (BPW, Difco). Appropriate dilutions were surface plated in duplicate on sorbitol MacConkey (SMAC) and on thin agar layer (TAL) medium (SMAC overlaid with 14 mL of TSAYE). The inoculated agar plates were incubated at 35 °C for 24 h (TAL) and 48 h (SMAC) before colonies were counted. In instances when the numbers of *E. coli* survivors were lower than the detection limit (10 CFU/mL) of the plating method, juice samples were enriched in TSBYE with added selective cefexime-tellurite (CT) supplement for 24 h and then streak plated on SMAC with CT supplement to determine the presence or absence of the pathogen [42].

### 2.6. Determination of Sub-Lethal Injury

For each type of agar medium (SMAC agar and TAL medium), the number of *E. coli* O157:H7 survivors after each HVACP treatment time were used to calculate the reduction factor (RF). For each treatment time, the RF was calculated by dividing the viable count (CFU/mL) of NAA or AA cells in the juice before HVACP treatment by the CFU/mL in the juice after treatment. The log (RF) is expressed as shown in the following equation [43]:

Log RF = log [CFU/mL before HVACP treatment **÷** CFU/mL after HVACP treatment]

For all treatment times (0, 1, 3, 5, and 7 min), the log RFs for the selective medium (SMAC; *y*-axis) versus the log RFs for the TAL medium (*x*-axis) were plotted. Linear regression lines were fitted through the data points, and sub-lethal injury was determined by comparing the slopes to 1 and the *y*-intercepts to 0 [43].

### 2.7. pH Evaluation of Pineapple Juice

The pH of the pineapple juice was measured using a pH meter (Accumet Basic AB15 pH meter, Thermo Fisher Scientific Inc., Waltham, MA, USA). Prior to each measurement, the samples in the tubes were mixed by vortexing to prevent separation. For each replicate experiment, two pH measurements were performed on the control and treated juice samples.

### 2.8. Measurement of Degrees Brix of Pineapple Juice

The degrees Brix (°Brix) of the juice was measured using a refractometer (Atago PAL 1, Atago Co., Ltd., Tokyo, Japan). Distilled water was used to represent a blank liquid sample. Samples of pineapple juice in tubes were homogenized by vortexing, and a separate Pasteur pipette was used to add a small amount of each juice to the refractometer well for measurement. For each replicate experiment, two °Brix measurements were performed on the control and treated juice samples.

### 2.9. Statistical Analysis

For microbiological analysis and quality evaluation tests (pH, and Brix), at least three replications of each experiment were performed. JMP Pro statistical software version 16 (SAS Institute, Inc., Cary, NC) was used to analyze the average number of survivors as a function of HVACP treatment time. To determine significant differences, an analysis of variance (ANOVA) was performed with a *p*-value < 0.05. Tukey’s honestly significant difference (HSD) test was performed to identify the means that were significantly different from each other.

## 3. Results

### 3.1. Survivors of E. coli in Pineapple Juice at 0.1 h and 24 h after HVACP Treatment

**Figure 2:**
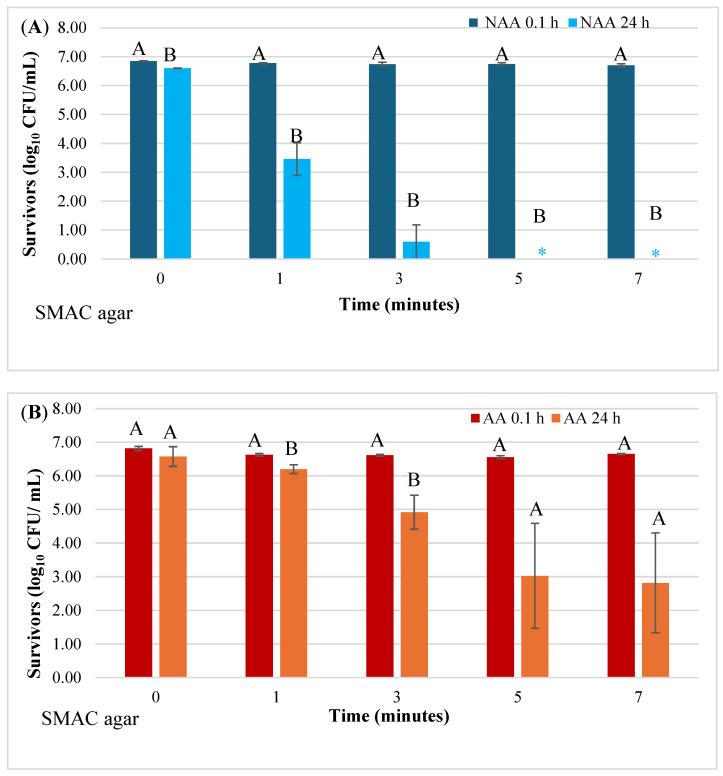
Effect of holding time (0.1 h and 24 h) following HVACP (70 kV) treatment on the viability of non-acid-adapted (**A**) and acid-adapted (**B**) *Escherichia coli* O157:H7 survivors in pineapple juice. The survivors were recovered on sorbitol MacConkey (SMAC) agar. For each treatment time, different letters (A or B) above the bars indicate a significant difference in the number of survivors at 0.1 h and 24 h post-treatment (*p* < 0.05). Asterisk (*) indicates that the pathogen was not detected.

Survivors of NAA and acid AA *E. coli* O157:H7 in pineapple juice, based on colony counts on SMAC agar, are shown in Figure 2A,B. When juice samples were analyzed at 0.1 h after HVACP treatment, viable counts of both NAA and AA cells did not differ from counts in control samples irrespective of treatment time (*p* > 0.05). Significant reductions in viable counts of both NAA and AA cells were observed in juice treated with HVACP for 1 and 3 min and held at 4 °C for 24 h (*p* < 0.05). However, the number of AA survivors exceeded that of NAA survivors. Following exposure to HVACP for 5 or 7 min and subsequently holding the juice at 4 °C for 24 h, the NAA cells in juice were completely inactivated (negative enrichment test) (Figure 2A). In contrast, the number of viable AA cells (log_10_ CFU/mL) in juice treated for 5 and 7 min and held at 4 °C for 24 h was 3.03 (5 min) and 2.82 (7 min) (Figure 2B).

**Figure 3:**
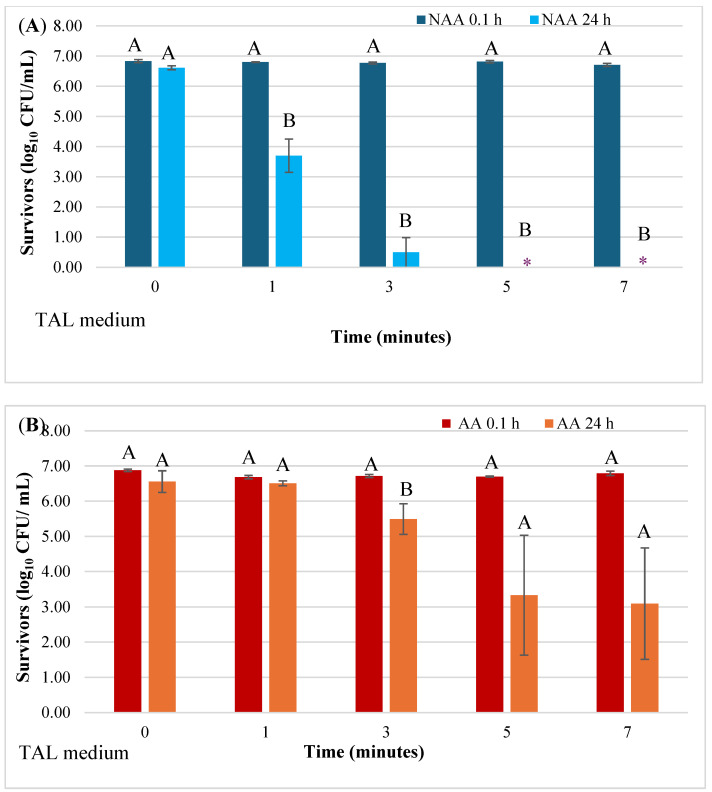
Effect of holding time (0.1 h and 24 h) following HVACP (70 kV) treatment on the viability of non-acid-adapted (**A**) and acid-adapted (**B**) *Escherichia coli* O157:H7 survivors in pineapple juice. Survivors were recovered on a thin agar layer (TAL) medium. For each treatment time, different letters (A or B) above the bars indicate a significant difference in the number of survivors at 0.1 h and 24 h post-treatment (*p* < 0.05). Asterisk (*) indicates that the pathogen was not detected.

As previously described for SMAC agar, a similar trend in viability of NAA and AA cells in control and HVACP-treated juice was observed based on bacterial counts on TAL medium (Figure 3A,B). Control and treated juice samples analyzed at 0.1 h after HVACP treatment exhibited high viable counts of both NAA and AA cells, with no significant differences among counts (*p* > 0.05). Significant decreases in NAA cells were evident in juices analyzed at 24 h, with no survivors detected in juice treated for 5 or 7 min (Figure 3A). Irrespective of the duration of HVACP treatment, AA cells of the pathogen were consistently detected in juice analyzed at 24 h (Figure 3B).

### 3.2. Effect of Physiological State on E. coli O157:H7 Tolerance of HVACP in Juice

**Figure 4:**
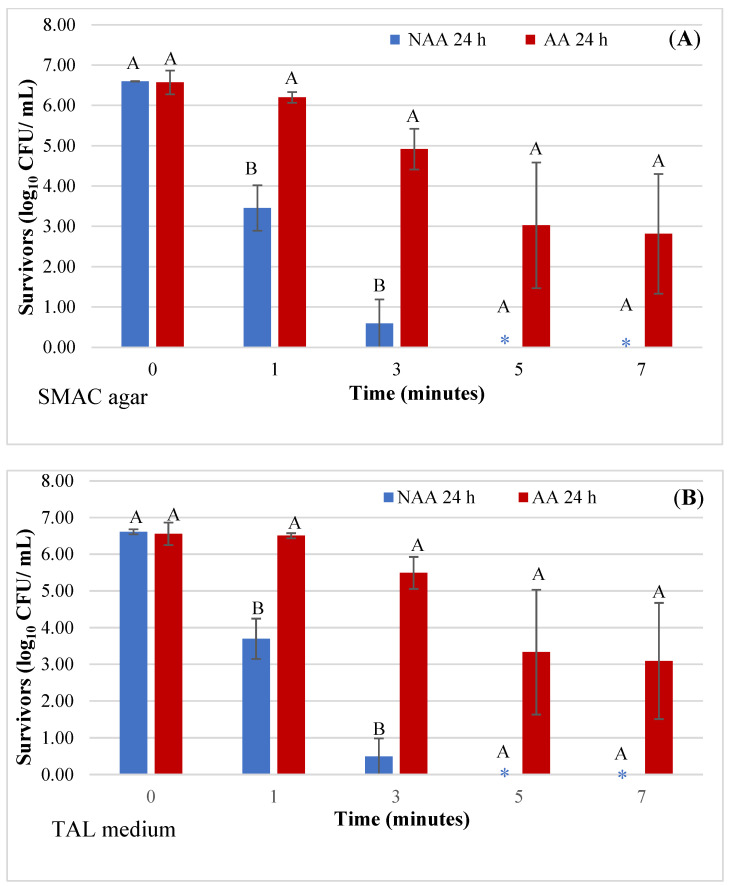
Effect of physiological state (NAA and AA) on the survival of *Escherichia coli* O157:H7 in raw pineapple juice held at 4 °C for 24 h after HVACP (70 kV) treatment. Survivors were recovered on sorbitol MacConkey (SMAC) agar (**A**) and TAL medium (**B**). For each treatment time, different first letters (A or B) above the bars indicate a significant difference in the viable counts of NAA and AA cells (*p* < 0.05). Asterisk (*) indicates that the pathogen was not detected.

A direct comparison of the effect of the physiological state (NAA and AA) on *E. coli* O157:H7 survivors in juice analyzed 24 h after HVACP treatment is shown in Figure 4A,B. Based on bacterial counts on SMAC agar (Figure 4A) and TAL medium (Figure 4B), the number of NAA and AA survivors in the control juice was not significantly different (*p* > 0.05). The number of NAA survivors in refrigerated juice treated with HVACP for 1 and 3 min was 3.46 and 0.59 log_10_ CFU/mL, respectively, whereas the number of AA survivors was higher at 6.51 (1 min) and 5.49 (3 min) (*p* < 0.05). Very similar results were observed when TAL medium was used to recover *E. coli* survivors (Figure 4B). For both recovery media, the number of NAA survivors of 5- and 7-min HVACP treatments was beyond detection (negative enrichment test); however, AA survivors were consistently detected with average viable counts ranging from 2.82 to 3.33 log_10_ CFU/mL (Figure 4A,B).

### 3.3. Sub-Lethal Injury of NAA and AA E. coli in Pineapple Juice

Sub-lethal injury (expressed by linear regression parameters) in NAA and AA *E. coli* O157:H7 survivors in HVACP-treated juice is shown in Table 1. Based on the method of Wuytack et al. [43], we observed that sub-lethal injury in NAA and AA survivors was not detected when juice samples were analyzed at 0.1 h after HVACP treatment. However, significant (*p* < 0.05) sub-lethal injury was detected in both NAA and AA survivors in juice analyzed 24 h following HVACP (70 kV) treatment (Table 1).

### 3.4. pH and Degrees Brix of Pineapple Juice

The effects of HVACP treatment on the pH and °Brix of pineapple juice analyzed at 0.1 h and 24 h after HVACP treatment are shown in Table 2. The initial pH of the pineapple juice (pH 3.36) decreased with increased exposure to HVACP; however, those decreases were not significant (*p* > 0.05). No differences were noted between the pH of the juice taken at 0.1 h and that taken at 24 h post-treatment (*p* > 0.05). A significant increase in °Brix was observed in juice that was exposed to HVACP for 3 or 7 min and tested at 0.1 h after treatment (*p* < 0.05). No increases in °Brix were observed in treated samples that were stored at 4 °C and tested after 24 h (*p* > 0.05). Juice evaluated after 24 h of storage (4 °C) had higher °Brix values compared to juice evaluated at 0.1 h after HVACP treatment (*p* < 0.05).

## 4. Discussion

### 4.1. Stress Adaptation in Foodborne Microorganisms

Foodborne microorganisms encounter a myriad of stressors in food processing environments, and survivors may become stress adapted, thus increasing their tolerance to subsequent chemical or physical food processes [44,45,46]. Typical stressors imposed by food processes include, but are not limited to, drying, high salt, shifts in temperatures, low water activity, exposure antimicrobial food preservatives, ultraviolet radiation, chemical cleaners and sanitizers, low pH, and organic acids [13,46,47,48]. Of those stressors, low pH and type of acid frequently impact bacterial survival and growth because organic acids and acid cleaners are widely used in the food industry. In this regard, foodborne bacteria have developed physiological mechanisms to enhance their survival by adapting to acid stress [13,47]. Moreover, acid adaptation in foodborne pathogens can cross-protect them against stress imposed by subsequently applied food processes [13,31]. Based on this concept, we hypothesized that AA *E*. *coli* O157:H7 cells are more tolerant than NAA cells of HVACP in pineapple juice.

### 4.2. Survivors of E. coli O157:H7 in Juice after HVACP Treatment

When juice samples were exposed to HVACP for 1 to 7 min and then transferred to nutrient agar within 0.1 h, no significant loss in the viability of NAA or AA *E. coli* O157:H7 occurred (Figure 2A,B and Figure 3A,B). By analyzing juice samples within such a short time after exposure to HVACP, it seemed that the treatments had no lethal effect on the pathogen. Our results are consistent with those of Hartanto [49], who reported significantly higher viable counts of *E. coli* O157:H7 in HVACP (80 kV)-treated organic pineapple juice analyzed within 1 h as opposed to 24 h after treatment. Our observation that HVACP-treated NAA and AA cells exhibited significantly lower viable counts in juice after 24 h suggests an increased sensitivity of those cells to the juice environment. This sensitivity is likely due to sub-lethal structural and/or metabolic injury initiated by HVACP that was exacerbated by inhibitory conditions in the juice. The hostile environment of the pineapple juice (pH 3.36) likely precluded cellular repair, caused further injury, and decreased *E. coli* O157:H7 survivors in the juice held at 4 °C for 24 h. Also, pineapple juice has phenolic compounds and organic acids, such as malic acid, citric acid, ascorbic acid, and isocitric acid [50,51], that can prevent repair processes in sub-lethally injured cells [35]. Moreover, Yadav and Roopesh [52] demonstrated synergistically higher bacterial inactivation resulting from combining ACP and organic acids.

The results of the present study further suggest that sub-lethal injuries in both NAA and AA cells were repaired when cells were transferred to nutritious agar media within 0.1 h after HACP treatment. This may explain our earlier mentioned observation of no significant loss of viability of NAA cells in HVACP-treated juice, which was plated on agar media within 0.1 h after treatment.

### 4.3. Effect of Physiological States on the Number of E. coli Survivors

Figure 4A,B shows the results of a direct comparison of the effects of physiological state (NAA vs. AA) on the number of *E. coli* O157:H7 survivors in HVACP-treated juice for 24 h (Figure 4A,B). For all treatment times (1 to 7 min), irrespective of the type of recovery medium, the number of AA survivors in juice was significantly higher (*p* < 0.05) than that of NAA survivors. A very low number of NAA cells (0.49 log_10_ CFU/mL) survived the 3.0-min HVACP treatment of juice (Figure 4B). This result demonstrates a greater than 6.0 log_10_ CFU/mL reduction of the initial viable count of the NAA pathogen. Based on the use of NAA cells, the HVACP (70 kV, 3 min) process would fully comply with the FDA juice HACCP regulation requiring a 5.0 log_10_ CFU/mL reduction of the pertinent pathogen [12]. In contrast, the high numbers of AA cells (5.49 log_10_ CFU/mL) that survived those same processing parameters represented only a 1.03 log_10_ CFU/mL reduction (Figure 4B). Considering that both NAA and AA cells in the control (0 kV) juice survived in high numbers (6.56 to 6.61 log_10_ CFU/mL) for 24 h, the very low numbers of NAA in HVACP-treated juice suggest that AA *E. coli* O157:H7 was more tolerant to HVACP. Therefore, to ensure the microbial safety of acidic juices treated with cold plasma technology, AA cells of the pertinent pathogen should be used in process validation studies.

Tosun and Gonul [53] demonstrated that acid shock of *E. coli* O157:H7 for 1 h in TSB (pH 4.5 to 5.5) significantly increased its acid tolerance in TSB at pH 2.5 to 3.0. In the present study, there was a 1 h interval between inoculation of the juice samples with the pathogen and HVACP treatment. Therefore, the similarity in survival of NAA and AA cells in the control juice is likely due to NAA cells triggering an acid shock response [54], which protected them against the acidic conditions in the juice. Unlike the NAA cells, far higher numbers of AA cells that survived after 24 h in treated juice (Figure 4A,B) suggest that acid adaptation exerted a protective effect against HVACP. Wang et al. [47] reported that acid adaptation, apart from inducing bacterial tolerance to low pH, may also trigger physiological and genetic systems that aid in cross-protecting cells against different stressors.

In the present study, the short HVACP treatment time and refrigerated storage caused significantly higher inactivation of NAA cells compared to AA cells (Figure 4A,B). Although the exact mechanism of microbial inactivation by ACP is unclear, it involves oxidative stress caused by ROS [21,48]. Extensive oxidative stress in *E*. *coli* cells with DNA and membrane damage caused by DBD ACP can lead to cell death [48]. Hu et al. [48] reported that the level of intracellular ROS was significantly lower in AA compared to NAA cells of *S*. *enteritidis*. Yuk and Marchall [30] demonstrated that AA *E. coli* O157:H7 had a higher level of saturated fatty acids in the membrane compared to NAA cells. A higher level of saturated fatty acids could increase bacterial tolerance to oxidative attack by ROS [29]. Therefore, based on those previously stated findings, the ACP sensitivity of NAA cells is likely due to: (i) more accumulation of intracellular ROS in NAA cells due to their relatively lower ability to scavenge ROS, and/or (ii) a lower concentration of saturated fatty acids in their cell membrane that decreased membrane resistance to oxidative damage. Moreover, the acidic pH (pH 3.36) of the pineapple juice as well as the refrigeration temperature (4 °C) used in the present study likely caused additional stress on the HVACP-damaged NAA cells, which resulted in their inactivation.

Acid adaptation can be induced by exposure of microbial cells to a gradual decrease in pH. This situation simulates environmental stress conditions in a fermented product in which acids are gradually produced by the natural microflora or by acid-producing bacteria that are added to the food products. In the present study, we cultured *E. coli* O157:H7 in TSB (with 7.5 g dextrose added) at 35 °C for 24 h to obtain AA cells [29]. Our results are consistent with those of Liao et al. [26], who reported that long-term (24 h) acid adaptation of *Staphylococcus aureus* at pH 4.5 resulted in its higher tolerance to cold plasma compared to NAA cells of that same organism. These findings are inconsistent with results obtained from short-term acid adaptation (4 h) of *S. aureus*, which exhibited no increased tolerance to the plasma [33]. Similarly, short-term (2 h) acid adaptation induced with different acids, including hydrochloric, ascorbic, acetic, citric, lactic, and malic at pH (6.4, 5.4, and 4.5), had very little effect on the tolerance of *Salmonella* Typhimurium and *Salmonella* Enteritidis to cold plasma in a model system [15]. Based on the research findings and results of the present study, it seems that foodborne bacteria require prolonged exposure to low pH conditions to develop cross-protection against cold plasma.

### 4.4. Sub-Lethal Injury of NAA and AA E. coli in Pineapple Juice

To determine sub-lethal injury in surviving populations of NAA and AA *E. coli* O157:H7, we used the method described by Wuytack et al. [43], which is based on the reduction factor (RF) concept as previously described in Section 2.7. For each treatment time, the Log RF for the selective medium (SMAC; *y*-axis) versus the Log RF for the TAL medium (*x*-axis) were plotted. Linear regression lines through the data points were used to determine sub-lethal injury by comparing the slopes to 1 and the y-intercepts to 0 [43]. There is no sub-lethal injury when the slope of the line is 1.0 and the intercept is 0. In this instance, the same decrease in viability occurred on the SMAC agar and on the TAL medium. A slope that is significantly greater than 1.0 or an intercept that is significantly greater than 0 suggests sub-lethal injury (*p* < 0.05). This is based on the larger viability reduction on SMAC agar than on the TAL medium. Moreover, one can assume that a larger deviation of the slope from 1.0 or a larger deviation of the intercept from 0 reflects a greater extent of sub-lethal injury.

Our results showed that the intercept of the regression line for viability reduction in NAA and AA *E. coli* O157:H7 in HVACP-treated pineapple juice held at 4 °C for 24 h was significantly larger than 0 (Table 1). These results suggest that sub-lethal injury occurred among NAA and AA survivors in juice held at 4 °C for 24 h but was not detected in NAA and AA survivors in juice samples that were plated within 0.1 h after HVACP treatment.

A factor that likely caused sub-lethal injury in NAA and AA survivors in the juice is the residual reactive species generated by cold plasma. Short-lived reactive species, such as free electrons (e^−^), singlet oxygen (O), hydrogen atom (H), hydroxyl radical (OH^•^), hydroperoxyl radical (HOO^•^), and superoxide anion (O_2_^−^) impinge on the surface of liquids and react with water molecules to form more long-lived secondary species [55,56]. Long-lived secondary species such as ozone (O_3_), hydrogen peroxide (H_2_O_2_) nitrous acid (HNO_2_), and peroxynitrous acid [57] continue to exert an antimicrobial effect even after HVACP treatment has stopped [58]. Han et al. [24] published scanning electron microscopy images of *S. aureus* and *E. coli* cells exposed to cold plasma treatment at 80 kV and held for 24 h after treatment. The cells treated with indirect plasma showed signs of structural damage in comparison to their non-treated counterparts, indicating damage via residual reactive oxygen and nitrogen species. All of these species can cause further lesions and prevent the resuscitation of sub-lethally injured bacteria [35,59]. Therefore, in the present study, the stress imposed by residual long lived plasma species and the intrinsic factors of pineapple juice (i.e., low pH, organic acids, phenolic compounds) contributed to sub-lethal injury in the NAA and AA *E. coli* cells recovered from the juice held at 4 °C for 24 h.

The determination of sub-lethal injury in foodborne pathogens after their exposure to chemical or physical food processes is important for two main reasons: (i) failure to detect sub-lethally injured pathogens in a processed food or beverage product can overestimate the antimicrobial effectiveness of the applied process. This is also important when selective media are utilized to recover pathogen survivors, because, depending on the extent of injury, sub-lethally injured cells may not grow on selective media. (ii) Levels of antimicrobial treatments that inflict sub-lethal injury in pathogens provide opportunities for their use in combination with other interventions to prevent cellular repair and ultimately inactivate those pathogens.

### 4.5. pH and Degrees Brix of Pineapple Juice

The pH and Brix values of juice treated with HVACP (Table 2) are similar to those stated in the published literature on pineapple juice [51]. Our results show a slight decrease in the pH of the juice with increased exposure to HVACP; however, it was not significantly different from the pH of the control juice (Table 2). Several studies that used the DBD method for applying cold plasma to various juices demonstrated that pH decreased with increasing exposure to HVACP [32,39,49]. One likely reason for the decrease in pH is the formation of nitrous acid (HNO_2_) or nitric acid (HNO_3_) generated from reactive nitrogen species during and after HVACP treatment [60].

Our results indicate that HVACP significantly increased the Brix of the juice samples, which were analyzed within 0.1 h after treatment (*p* < 0.05). Both Hartanto et al. [49] and Liao et al. [39] also found that increasing HVACP treatment times increased the Brix of juice. The increase in Brix could be attributed to ROS such as ozone causing the depolymerization of polysaccharides via oxidation, thus creating shorter chain polysaccharides and smaller soluble units (sugars). Additionally, ozone species could cleave the glycosidic bonds and oxidize the functional groups of polysaccharides, leading to the development of lactones, carbon dioxide, hydroperoxides, carbonyl, and carboxyl compounds [61,62]. The stabilization of the Brix in juice analyzed after 24 h is likely due to the completion of further depolymerization reaction by residual reactive plasma species during the 24-h refrigeration (4 °C) of the juice.

## 5. Conclusions

Direct application of HVACP (70 kV) for 3, 5, or 7 min significantly reduces populations of NAA *E. coli* O157:H7 by greater than 5.00 log_10_ CFU/mL in pineapple juice during post-treatment storage (4 °C) for 24 h. However, those same HVACP treatments yield significantly lower reductions of AA *E. coli* that fail to comply with the FDA juice HACCP regulation for killing *E. coli* O157:H7. Acid adaptation can cross-protect *E. coli* against the bactericidal effect of HVACP in pineapple juice and should be considered when designing protocols to ensure the microbial safety of juices treated with cold plasma technology.

## Figures and Tables

**Table 1:** Sub-lethal injury (based on linear regression parameters *) in survivors of non-acid-adapted (NAA) and acid-adapted (AA) *Escherichia coli* O157:H7 in pineapple juice treated with HVACP (70 kV) and recovered at 0.1 h and 24 h after HVACP treatment.

Physiological State	Time	Slope	y-Intercept	R^2^
NAA	0.1 h	0.06 ± 0.55	0.056 ± 0.05	0.154 ± 0.097
NAA	24 h	0.983 ± 0.01	0.093 ± 0.03 ^a^	0.997 ± 0.002
AA	0.1 h	0.73 ± 0.51	0.063 ± 0.05	0.417 ± 0.33
AA	24 h	1.01 ± 0.12	0.28 ± 0.02 ^a^	0.942 ± 0.07

* Values are averages ± standard deviations from three replications of the experiment; ^a^ Intercept significantly different from 0 (*p* < 0.05).

**Table 2:** The pH and degrees Brix of pineapple juice at 0.1 h and 24 h after treatment with HVACP (70 kV).

	pH		Brix	
Time (min)	pH; 0.1 h	pH; 24 h	Brix; 0.1 h	Brix; 24 h
0	3.36 ± 0.03 A,x	3.36 ± 0.03 A,x	14.3 ± 0.00 A,x	14.5 ± 0.00 A,y
3	3.35 ± 0.04 A,x	3.35 ± 0.02 A,x	14.4 ± 0.00 B,x	14.5 ± 0.00 A,y
7	3.33 ± 0.01 A,x	3.34 ± 0.01 A,x	14.4 ± 0.00 B,x	14.5 ± 0.00 A,y

Values are averages ± standard deviations from four replications of the experiment. Different uppercase letters (A, B) within a column indicate significant differences in pH or °Brix (*p* < 0.05). Different lowercase letters (x, y) within a row indicate significant differences (*p* < 0.05) for pH or °Brix taken at 0.1 h or 24 h at specific treatment times (0, 3, or 7 min).

## Data Availability

The raw data supporting the conclusions of this article will be made available by the authors on request.
